# Multicellular 3D Models for the Study of Cardiac Fibrosis

**DOI:** 10.3390/ijms231911642

**Published:** 2022-10-01

**Authors:** Vittorio Picchio, Erica Floris, Yuriy Derevyanchuk, Claudia Cozzolino, Elisa Messina, Francesca Pagano, Isotta Chimenti, Roberto Gaetani

**Affiliations:** 1 Department of Medical Surgical Sciences and Biotechnologies, Sapienza University, 04100 Latina, Italy; 2 Department of Molecular Medicine, Sapienza University, 00161 Rome, Italy; 3 Institute of Biochemistry and Cell Biology, National Council of Research (IBBC-CNR), 00015 Monterotondo, Italy; 4 Mediterranea Cardiocentro, 80122 Napoli, Italy

**Keywords:** cardiac fibrosis, 3D cultures, cardiac stromal cells, cardiac fibroblasts, organoids, organs-on-chip, tissue engineering

## Abstract

Ex vivo modelling systems for cardiovascular research are becoming increasingly important in reducing lab animal use and boosting personalized medicine approaches. Integrating multiple cell types in complex setups adds a higher level of significance to the models, simulating the intricate intercellular communication of the microenvironment in vivo. Cardiac fibrosis represents a key pathogenetic step in multiple cardiovascular diseases, such as ischemic and diabetic cardiomyopathies. Indeed, allowing inter-cellular interactions between cardiac stromal cells, endothelial cells, cardiomyocytes, and/or immune cells in dedicated systems could make ex vivo models of cardiac fibrosis even more relevant. Moreover, culture systems with 3D architectures further enrich the physiological significance of such in vitro models. In this review, we provide a summary of the multicellular 3D models for the study of cardiac fibrosis described in the literature, such as spontaneous microtissues, bioprinted constructs, engineered tissues, and organs-on-chip, discussing their advantages and limitations. Important discoveries on the physiopathology of cardiac fibrosis, as well as the screening of novel potential therapeutic molecules, have been reported thanks to these systems. Future developments will certainly increase their translational impact for understanding and modulating mechanisms of cardiac fibrosis even further.

## 1. Introduction

Cardiac fibrosis is characterized by an increased number of fibroblasts with enhanced deposition of extracellular matrix (ECM). It represents the bottleneck of several pathological processes, including ischemic/non-ischemic cardiomyopathy, metabolic syndrome, diabetes, and aging [1]. Fibrosis leads to loss of functional cardiomyocytes, increased stiffness associated with diastolic dysfunction, and impairment of electromechanical coupling [2].

The fibrotic process can be considered as a continuum of multiple attempts for healing/repair reactions, gradually evolving into a chronic degenerative/inflammatory response, and ending with fibrosis. The whole process involves myocytes as well as non-myocytic cells, such as stromal cells, endothelial cells, neuronal cells, and local macrophages. Cardiac injury induces a rapid proliferation and activation of primitive non-activated stromal cells and mature interspersed fibroblasts [3], with increased expression of collagens and cell cycle genes [4]. Part of the activated fibroblasts differentiate into myofibroblasts, express smooth muscle actin (SMA), and produce excessive ECM components, impairing cardiac function with time. The clinical outcome of this process is detectable as impaired cardiac function, and is highly dependent on the number and distribution of cells involved. Indeed, defined structural damages to the myocardium may not result in a detectable functional decline for many years due to the recovery of the tissue via compensatory mechanisms, until remodelling becomes very advanced. Conversely, mechanosensing-mediated damage (e.g., due to prolonged changes in haemodynamics, particularly arrhythmic processes) is associated with the subsequent development of changes in the cardiac structure.

It is well known that there is a tight connection between inflammatory and fibrotic pathways in the pathophysiology of tissue fibrosis and remodelling [5,6,7], corresponding also to a shift in the paracrine activity of stromal cells with production of pro-inflammatory and pro-fibrotic factors and cytokines [8,9,10,11].

In this regard, basic and translational research on cardiac physiopathology can take great advantage from innovative 3D cardiac in vitro models bringing together more than one cell type. Indeed, 3D models, from organoids to microfluidic platforms, could be particularly relevant for studies related to the secretome and inflammasome, enabling the generation of different types of complex microenvironments. These models are helpful for the study in a more realistic condition of many pathogenetic processes, including cardiac fibrosis. Moreover, they represent important translational models to discover and test new drugs and therapeutics, supporting the clinical translation of novel protocols in a “personalized medicine” perspective. Notwithstanding the importance of animal models in biomedical and clinical research, they often do not fully recapitulate the pathogenesis of human cardiac fibrosis. In addition, the 3Rs principle of replacement, reduction, and refinement encourages the progressive reduction of animal experimentation. Furthermore, the use of animal models entails housing and handling costs that cannot be ignored. Under this perspective, 3D cultures and innovative microfluidic devices represent useful platforms to perform significant investigations in vitro on multiple topics, including cardiovascular diseases.

This review presents an overview of innovative multi-cellular 3D models described in the literature to study cardiac fibrosis. Indeed, advancements in this kind of in vitro tissue models may open new pathways for disease modelling, drug discovery, and regenerative medicine by providing fast and controllable platforms with easy availability and relatively low cost compared to animal models.

## 2. Sometimes Three Dimensions Are Better than Two

**Figure 1:**
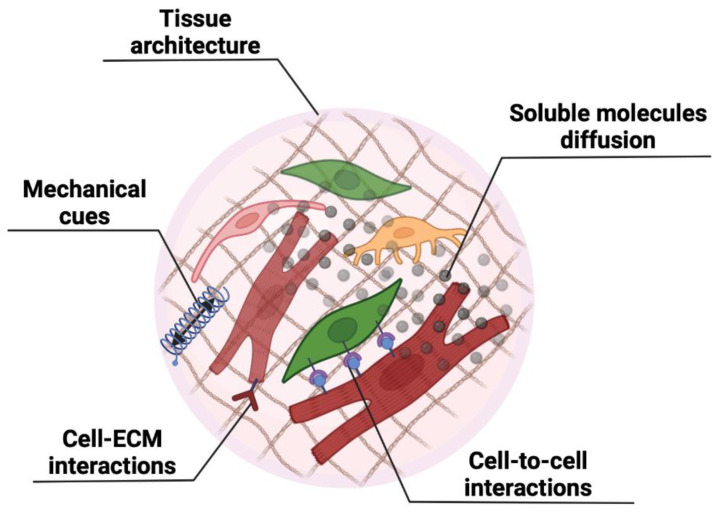
Summary of the main advantages of multicellularity and 3D architecture in modelling systems. Several features can be enhanced and boosted when co-culturing multiple cell types in 3D microenvironments. They include complex tissue architecture, the diffusion of paracrine soluble molecules, the establishment of cell-to-cell and cell–ECM interactions, and integrated mechanical sensing and responses. This figure was created with Biorender software.

Classical two-dimensional (2D) cell culture techniques have been always used as a standard model to evaluate cellular physiological mechanisms and response to different biochemical signals. However, in the last two decades, the development of new biomaterials, new technologies (such as tissue printing), and our better understanding of fundamental biological mechanisms has prompted many researchers to move to 3D culture approaches [12]. Regular 2D cultures have the advantage of providing homogeneous nutrient and growth factor supply to all cells, therefore limiting potential variability in the experimental readout. However, cells in the body are embedded in a 3D architecture, and their responsiveness and behaviour are controlled by multiple factors, such as soluble molecules, cell-to-cell contacts, cell–ECM contacts, and the mechanical cues coming from specific microenvironments [13] (Figure 1). The combination of these stimuli acts synergistically to provide the necessary signals regulating cellular functions in the developing and adult organism [14]. For example, cell–ECM interaction has been shown to be fundamental in cellular behaviour control. Cells can bind to the ECM through signal receptors which can induce a specific response, such as proliferation, differentiation, maturation, and migration [15].

A well-known example of cellular control by the microenvironment is the stem cell niche, present in various adult tissues [16]. Multiple signals within the niche architecture act together to determine the fate of stem and progenitor cells, and control cell differentiation based on physiological and pathological signals; these inputs regulate either physiological tissue homeostasis or the response to tissue damage [15]. Similarly, in artificial microenvironments it has been shown how cardiomyocyte maturation and differentiation from stem cells is enhanced when they are maintained as 3D embryoid or organoid bodies, as compared to 2D cultures [17,18]. Indeed, cardiac progenitor cells enhance their cardiogenic commitment when cultured in 3D as compared to the 2D culture condition [19,20]. Therefore, three dimensions should help when obtaining and preserving mature and representative cardiac phenotypes in vitro.

Cells also respond to different biomechanical stimuli which are then converted in biochemical signals through various mechanisms, ranging from actin-myosin contraction regulation to focal adhesion-based signalling mechanisms, or force-sensitive transcription factor localization and stretch-activated channels causing ion flux changes [13,21]. These responses are even more significant in 3D conditions (Figure 1). In tissue engineering applications, it has been demonstrated how substrate stiffness can control cell differentiation and maturation [22]. Recapitulating in vitro the mechanical stress at which the cells are exposed in vivo is of paramount importance for the generation of relevant models for both healthy and pathological tissues, as the mechanical properties of fibrotic tissues are significantly impaired. For example, when cardiac progenitor cells were cultured in a 3D hydrogel with tuneable mechanical properties, cells responded to the mechanical cues by increasing their migration and proliferation capabilities if cultured on softer gel, while increasing the elastic modulus of the hydrogel resulted in a higher expression of cardiomyocyte-related genes [23].

Multiple reports have used mechanical and electrical stimulation to advance tissue differentiation and maturation in 3D [24,25,26,27]. Mechanical stimuli have also been used to mimic pathological responses from the tissue. In a 3D model of cardiac fibrosis, a strain–response correlation of mechanical stimulation and cardiac fibroblast (CF) phenotype has been shown, providing insight on how mechanical stimuli were necessary to better mimic a fibrotic response in a 3D model of cardiac fibrosis [28]. A 3D culture system of cardiac stromal cells has also been exploited to show how blocking the YAP-mediated molecular transduction of mechanical signals can interfere with the fibrotic specification of these cells, with a positive effect on the progression of maladaptive fibrosis [29].

**Figure 2:**
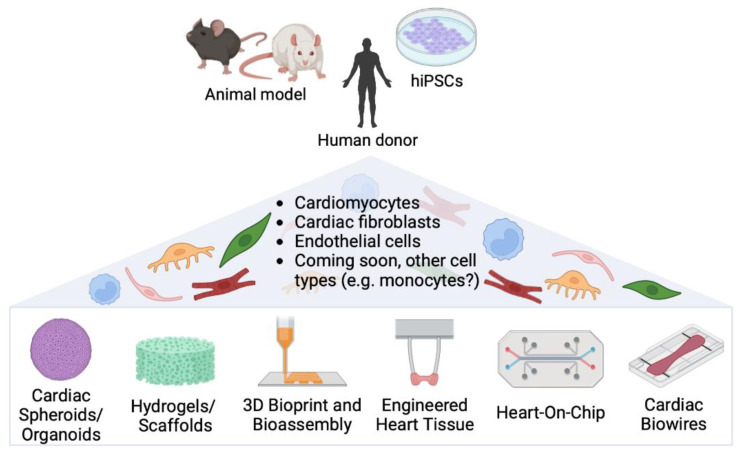
Multicellular 3D modelling systems of the cardiac microenvironment. Multiple cardiac cell types can be obtained from the tissue of animal models, human donors, or from differentiation of human induced pluripotent stem cells (hiPSCs). Multiple setups are depicted allowing culture in three dimensions to recapitulate the complex cardiac microenvironment. This figure was created with Biorender software.

In this context, it is clear how integrating the multiple signals to which cells are usually exposed in vivo in 3D models is indeed fundamental for the development of tissue-like structures that closely mimic healthy and pathological tissue (Figure 2). However, despite recent advances in biomaterial synthesis, the development of new biomimetic materials, and the development of complex bioreactors and devices for cell stimulation, many challenges still exist, such as the spatial–temporal control of cells within the 3D construct, standardization of oxygen and nutrient supply throughout the structure, generation of the right interface between different cell types, and media recipes when multiple cell types are used (Figure 1).

## 3. The More Cell Types, the Merrier

Multicellularity is a key feature of mammalian tissues, including the heart. Therefore, any attempt at accurate modelling of physiology or disease must consider the native complexity of the myocardium (Figure 1). Many approaches have been used through the years to define the proportion of the different cardiac cell types. Studies in the murine heart have reported 31% of nuclei (cumulatively from all cardiac regions) being cardiomyocytes, 43% being endothelial cells, and the remaining proportion encompassing various stromal and immune cell types [30]. More recently, single cell sequencing of the adult human heart has revealed that half of ventricular cells and nearly two thirds of atrial cells are non-cardiomyocytes, with a much lower proportion of endothelial cells compared to other stromal types (e.g., fibroblasts, pericytes) [31], and compared to the murine heart. Therefore, species-specific considerations may be also necessary based on the target of the modelling strategy.

Until recent years, many efforts in modelling cardiac homeostasis and disease have mainly focused on parenchymal cells, that is, cardiomyocytes. This could be sufficient for the study of features related to contractility and electrophysiology, although even for these advanced functions single-cell cultures can be sometimes reductive. As an example, inclusion of stromal cells can help tissue modelling protocols to reach a more advanced differentiation state and render them more performing [32,33,34], thus more relevant also for translational studies, such as drug testing. This effect derives, among others, from the beneficial paracrine effects of stromal cells towards cardiomyocytes [35,36], including the important contribution of stromal cells to electrical conductance through connexin-43 (CX43) expression [32] and to the specific differentiation of cardiomyocytes of the conduction systems [37]. Functional electrophysiological cues were precisely discovered thanks to multicellular modelling itself [38]. Furthermore, it has been known for some time now that multicellularity contributes to an overall more accurate resemblance of native mechanical features of the myocardial tissue as a whole [39]. This becomes particularly important when the pathological condition to be mimicked is characterized by changes in the cell type composition and biomechanical features of the tissue, as we will further discuss below.

CFs are important cells for tissue homeostasis. They support ECM maintenance and angiogenesis, and exert many paracrine functions for the benefit of cardiomyocytes, vessels, and immune cells [3,35,40]. They are the main players in fibrotic processes, thus making them essential components for in vitro modelling of cardiac fibrosis of any aetiology. Their activation after an acute insult, or during chronic cardiac damage, changes their phenotype [41,42]. In fact, they differentiate into functional states with enhanced ECM deposition capacity (e.g., myofibroblasts), and this phenotypic change is one of the foundations of cardiac fibrosis, remodelling, and stiffening [5,21,35]. Besides qualitative changes in their phenotype, CFs become more abundant during tissue repair and fibrosis. Therefore, modelling cardiac fibrosis cannot ignore the importance of resembling the specific cell-type composition in the diseased myocardium. For example, reducing the relative proportion between cardiomyocytes and fibroblasts (from 1:1 to 1:3, respectively) can very well mimic the fibrotic myocardium [43]. But why does multicellularity remain of great importance for the study of cardiac fibrosis, if it is mainly mediated by one cell type? Because many mechanisms contributing to stromal cell activation depend on other cell types. For example, cardiomyocyte stress and death activate inflammatory fibrosis through different pathways. Release of intracellular molecules by injured tissues (damage-associated molecular patterns—DAMPs) can change the paracrine behaviour of surrounding cells [44], thus mimicking more accurately the microenvironment sustaining fibrotic mechanisms in stromal cells (Figure 1). Moreover, detrimental changes in cardiomyocyte metabolism and oxidative state can contribute to increased oxidative stress within the myocardium. Reactive Oxygen Species can then directly activate stromal cells through STAT3 [42], further increasing their fibrotic commitment.

For the recent scientific effort towards the generation of complex systems which model organs physiology and pathology, and the high impact of the topic in future biomedicine applications, we will review and discuss advanced 3D models with a multi-cellular composition.

## 4. Microtissues: Cardiac Spheroids and Organoids

Three-dimensional culture systems offer multiple advantages for in vitro phenotype control to obtain physiologically relevant settings. The simplest 3D culture system is represented by spheroids, which can be obtained from different cell types (both somatic and stem/progenitor cells), including resident cardiac stromal cells [45,46]. Cardiac cell spheroids, despite their simplicity, can provide useful preliminary models even for the study of complex pathological issues like tissue stiffening and fibrosis [29,47].

A more complex example of 3D microtissue is represented by organoids [48]. Cardiac organoids are self-assembling structures of cardiac cell types mostly obtained from the proliferation and differentiation of multi/pluripotent stem cells. These structures replicate cell–cell interaction, cell–ECM interaction, and organ architecture and function at the microscale level, as similar as possible to in vivo histological features [49]. The formation process requires appropriate timing (sometimes weeks), and generally does not need specific equipment. Cardiac organoids can be formed from small cell numbers and usually grow up to 300 µm in diameter, so that oxygen and nutrients can still reach the deepest layers of the microtissue. They can be used as models of both physiological and pathological settings.

**Figure 3:**
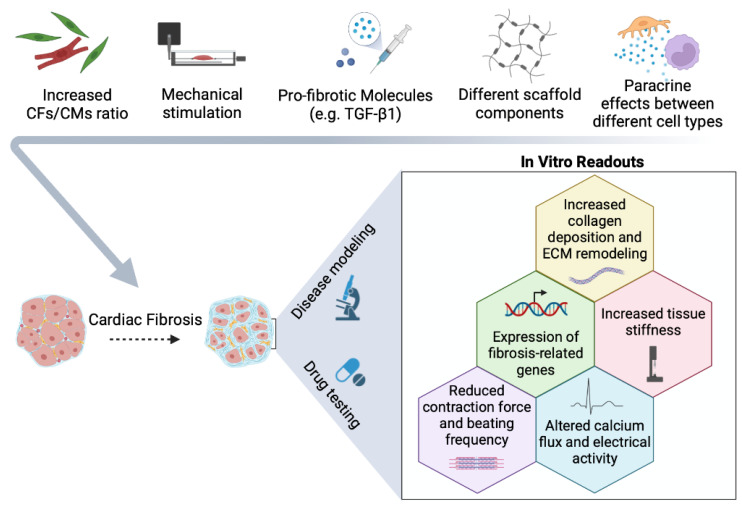
Tuneable parameters for simulating cardiac fibrosis. The application of different stimuli (e.g., pro-fibrotic molecules, mechanical stress, and variation of cellular composition) in multicellular 3D models allow features of cardiac fibrosis to be mimicked in vitro, which can be assessed and quantified by multiple in vitro readouts. Cardiac fibroblasts (CFs); cardiomyocytes (CMs). This figure was created with Biorender software.

In the field of cardiac microtissues, Figtree and colleagues have developed an elaborate 3D model, called vascularized cardiac spheroids (VCSs), obtained through the hanging drop co-culture of primary cardiac myocytes, CFs, and endothelial cells from neonatal rats. The stimulation of such VCSs with TGF-β1 (the most potent known pro-fibrotic factor) recapitulates typical features of the fibrotic process, including the increased deposition of ECM proteins (Figure 3). They have also been exploited to study fibrosis due to cardiotoxicity mechanisms: in fact, doxorubicin treatment induces pro-fibrotic features in VCSs, proving their versatility as cardiac fibrosis models of multiple aetiologies [50].

Cardiac microtissues offer the possibility to study cardiac fibrosis by modulating many of its characteristics. For example, it has been reported that increasing the fibroblast-to-cardiomyocyte ratio over 50% leads to increased collagen I deposition (Figure 3), yielding reduced contraction force and beating frequency in engineered cardiac microtissues, thus recalling the cardiac dysfunction consequent to fibrosis [51]. Another important parameter to consider when mimicking cardiac fibrosis in vitro is the mechanic microenvironment. Mainardi et al. assessed a micro-physiological system where several 3D cardiac microtissues can be seeded simultaneously and undergo cyclic stretching. The use of this device allows the investigation of cardiac fibrosis mechanisms induced by direct mechanical stimuli, without the requirement of pharmacological factors or stiff substrates. Again, an increased fibroblast-to-cardiomyocyte ratio leads to the outcome of fibrotic specification [52] (Figure 3).

Besides primary cell cultures, differentiated cardiac cells can be obtained from both human induced pluripotent stem cells (hiPSCs) and human embryonic stem cells (hESCs). Multi-cellular 3D microtissues can be composed of hESC-derived cardiomyocytes co-cultured with mesenchymal stem cells (MSCs), which can also be differentiated into fibroblasts. MSCs and CFs are shown to better recapitulate cardiac tissue organization and electrophysiological microenvironment, and TGF-β1 stimulation induces myofibroblast differentiation and a fibrotic phenotype of this 3D in vitro model [53]. Cardiac microtissues have also been used in the modelling of cardiac fibrosis consequent to an infarction injury. In detail, a 3D cardiac infarct organoid has been proposed by Richards et al., in which hiPSC-derived cardiomyocytes (iPSC-CMs), human CFs, human umbilical vein endothelial cells (HUVECs), and human adipose-derived stem cells (hADSCs) were cultured under hypoxia and norepinephrine stimulation in order to mimic myocardial infarction (MI) in vitro [54]. As a model of cardiac fibrosis, this “infarct organoid” showed the expression of many fibrosis-related genes and the presence of myofibroblast-like cells, as well as an increase in microtissue stiffness.

Overall, these 3D self-assembling systems resemble numerous pathogenetic features of cardiac fibrosis, but still show many limitations in their application as models, such as lack of integration of immune cells and other supporting cells. These features need to be implemented to reach higher levels of physiological relevance for cardiovascular disease modelling.

## 5. Bioprinting and Bioassembling

The bioprinting technique could be defined as computerized additive biofabrication of 3D cellular constructs. To date, many artificial tissue-like constructs, such as bone, vasculature, neural tissue, and cardiac muscle, have been created starting from autologous and patient-specific primary or stem cells [55,56]. Large components of cardiac tissues (such as tri-leaflet heart valves, or multiscale vasculature networks) can be produced rapidly and in significant numbers through 3D bioprinting based on bioinks composed of collagen and cardiac cell types, such as hESC-CMs or HUVECs [57] (Figure 2). The challenge, however, is to minimize cell stress during printing and provide close-to-native cardiac ECM. The recipe for the bioink, which is the foundation of the entire process, has to be biocompatible, as well as mechanically and structurally stable [58].

According to their base materials, bioinks can be divided into two principal groups: scaffold-based and scaffold-free bioinks. Scaffold-free bioprinting provides better biocompatibility for specific applications, such as transplantation to the heart [59]. On the other side, cells in the scaffold-free bioinks are printed without matrix support, and the ability to deposit their own ECM is encouraged to confer support for intercellular communication and proliferation to create strands or spheroids [60]. Compared to microtissues, bioprinting has the advantage that complex tissue architectures with specific cellular organization can be designed and created. Although cells for bioprinting can be added to the construct individually, often they are first preassembled as a whole, so that they produce their own ECM, giving the construct greater stability. Unfortunately, at least for the moment, the high costs and the complexity of the printing systems make this technology not easily accessible.

An example of a scaffold-free model is the biomaterial-free 3D-printed cardiac patch developed by Ong and colleagues. In this method, human iPSC-CMs, fibroblasts, and endothelial cells are first plated in ultra-low attachment 96-well plates to form spheroids, then the obtained spheroids are bioprinted, allocating them closely in the desired structure, allowing them to spontaneously fuse and form a continuous cardiac tissue [61]. Similarly, Daly et al. have developed a bioprinted cardiac tissue model obtained by seeding pre-formed spheroids into self-healing support hydrogels, leading to the assembly of high-cell-density microtissue with specific spatial organization. Spheroids are formed by co-culturing different ratios of human CFs and hiPSC–CMs, to recapitulate both healthy and fibrotic cardiac tissue (Figure 3). The spheroids are then loaded through 3D bioprinting, following a specific spatial distribution which mimics the presence of one or multiple scars within the artificial tissue, as well as “border zone” interactions. This system could allow in vitro drug testing in a clinically relevant tissue architecture [62].

Mechanical stimulation can also be modelled through 3D bioprinting by combining different hydrogel bioinks (Figure 3). Specifically, Shin and colleagues have developed bioinks composed of partially digested porcine cardiac decellularized ECM, Laponite-XLG nanoclay, and poly(ethylene glycol)-diacrylate (PEG-DA). This formulation was used to co-culture human CFs, hiPSC–CMs, and human bone marrow stromal cells, producing exciting bioprinting results in terms of shape fidelity, cross-linking, and cytocompatibility. Authors have proven that by changing the PEG-DA concentration of the hydrogel, it is possible to tune mechanical stiffness, ranging from that of healthy to fibrotic myocardium [63].

A different bio-assembling technology is the generation of spheroids through magnetic 3D bio-printing; this procedure consists of labelling cells with magnetic nanoparticles and assembling them into 3D structures under the guide of a magnet [64]. In this field, to the best of our knowledge, only one 3D cardiac construct has been developed: primary rat cardiomyocytes, fibroblasts, and endothelial cells have been assembled into multicellular cardiac aggregates using magnetic levitation [65]. Despite the usefulness of this 3D system, it has not yet been employed in the in vitro modelling of cardiac fibrosis.

## 6. Engineered Heart Tissues

Engineered heart tissues (EHTs) represent an advanced in vitro model for drug testing, disease modelling, and regenerative medicine. The first EHT was developed by the Eschenhagen group using neonatal rat cardiomyocytes [66]. EHTs have longitudinal orientation, and are characterized by intercellular coupling and force generation. EHTs based on hydrogels, where cardiac cells self-organize forming a tissue bundle around two elastomeric pillars, are now widely used. The two pillars exert a cyclic mechanical strain that promotes cell alignment and auxotonic contraction of the tissue. The deflection of the pillars gives an optical readout of contractile parameters and allows force measurements [67]. EHTs allow the study of the role of various cell types, besides cardiomyocytes and fibroblasts, in cardiac tissue homeostasis and fibrosis (Figure 2). For example, Szepes et al. have developed a Bioartificial Cardiac Tissue (BCT) to model in vitro myocardial interstitial fibrosis. In detail, the interplay of hiPSC–CMs, CFs, endothelial cells, and pericytes was investigated, revealing the emerging role of pericytes in tissue organization, mechanics, and remodelling. The inclusion of hiPSC-derived pericytes in place of fibroblasts led to increased BCT stiffness, similar to that of fibrotic cardiac tissue, due to higher collagen deposition and expression of many fibrosis-related genes [68] (Figure 3).

A key role in regulating cardiac tissue homeostasis and the fibrotic process is also played by immune cells, particularly resident macrophages. In fact, it has been already described that macrophage infiltration and expansion aggravates heart failure progression since macrophages produce pro-fibrotic factors, such as TGF-β1, PDGF, and Angiotensin II [69]. In addition, macrophage proliferation is stimulated by increased tissue stiffness, thus feeding a continuous positive feedback loop on cardiac fibrosis progression [70] (Figure 3). Given these findings, the introduction of macrophages into EHT systems appears to offer desirable progress [71], and could represent a great advancement in the ability to recapitulate the complexity of tissue microenvironments when modelling cardiac fibrosis.

Overall, EHTs represent potent models to be exploited, since they allow the integration of multiple cell types in the construct, in combination with electromechanical stimulation and readouts. Some limitations to their extensive use must be mentioned, however. First, the extremely high number of cells required makes them difficult to upscale and to subject to standardized quality control. Moreover, they require highly specialized operators and imaging techniques, thus still limiting their use to some extent.

## 7. Heart-on-Chips and Cardiac Biowires

In the last decade, bioengineering approaches have been integrated with microfluidic and microfabricated substrates, leading to the development of “organs-on-chip” [72]. These devices allow fine tuning of the geometry of the microenvironment and of media composition, as well as cell–cell interactions. The combination of cardiac cells and micro/nanoengineering devices has resulted in new in vitro models for the study of therapeutic approaches in cardiovascular diseases [73]. Heart-on-chip models can recapitulate typical features of the parenchymal structure of the myocardium, and primary physiological or pathological conditions of the human heart microenvironment.

Among the wide range of various heart-on-chip models, Biomimetic Cardiac Tissue Chips are composed of 3D fibres in which cardiomyocytes and CFs can be seeded, and haemodynamically stimulated to mimic volume and pressure changes occurring in the pumping heart. Stimulation under pressure overload for 48 hours in this device induces a fibrotic phenotype, as cardiac cells increase collagen deposition, the production of TGF-β1 and ECM remodelling factors [74] (Figure 3).

A cardiac-fibrosis-on-chip model has been also proposed, in which TGF-β1-stimulated human CFs and hiPSC–CMs are co-cultured. Fibrosis is simulated as increased deposition of ECM proteins and stiffness are detected; in addition, this fibrotic model reveals higher passive tension levels and reduced contraction force compared to control chips, as well as a different expression of several microRNAs involved in cardiac dysfunction and fibrosis (Figure 3). Therefore, this versatile cardiac-fibrosis-on-chip model can serve as a valid platform for anti-fibrotic drug testing with multiple readouts [75].

On the verge of modelling yet another aetiology, Veldhuizen et al. have reported the development of an ischemia-on-chip microfluidic model composed of a collagen-based hydrogel, cardiomyocytes, and CFs, that can recapitulate post-ischemia cardiac fibrosis through exposure to a controlled hypoxic environment [76].

More sophisticated heart-on-chip models include the so-called cardiac Biowires (Figure 2). Wang and colleagues have optimized a myocardial fibrosis platform called Biowire II, in which cardiac cylindrical tissue is suspended between poly(octamethylene maleate(anhydride)citrate) (POMaC) wires. This model exploits wire deflection to measure the contractility, active force, passive tension, and electrical properties of the artificial tissue. Human iPSC–CMs and CFs are seeded on a fibrin/Matrigel hydrogel. They can be mixed in different ratios to mimic healthy or fibrotic tissue (e.g., with increased collagen content), with consequent deterioration of contractile function and electrophysiological properties (Figure 3). The Biowire II model can also be integrated with heteropolar wires that allow the coupling of fibrotic and healthy tissue, thus recapitulating the interaction between a fibrotic region and the adjacent healthy tissue (such as the infarct border zone). Finally, these authors have proven the feasibility of the Biowire II as an anti-fibrotic drug screening platform, assessing the ability of several molecules to attenuate collagen deposition and passive tension [43].

Biowires have also been used to study integrative fibrosis-modelling strategies. Specifically, Kuzmanov et al. have analysed the proteomic profile of fibrotic cardiac tissues from different sources, including an engineered tissue composed of hiPSC–CMs and human ventricular fibroblasts seeded in different ratios on POMaC wires. This model seems to be an effective fibrosis-mimicking and drug testing platform with tight control over multiple parameters simultaneously [77].

Overall, the above-described bioengineering strategies to create models for cardiac fibrosis all require very advanced expertise and facilities, thus still hindering their wide dissemination. Nonetheless, they offer unique opportunities to analyse highly advanced features beyond biological readouts, including mechanical features and responses.

## 8. 3D Systems for Advanced Electromechanical Assessment

As mentioned before, the progression of fibrosis implies structural changes in the heart due to fibroblast activation and their differentiation into myofibroblasts. This process leads to the deposition of new ECM leading to variation in the intensity and direction of both contractile forces and electrical conduction. These changes produce electrophysiological abnormalities and biophysical modifications, such as enhanced tissue stiffness and compaction [69]. Moreover, the activation and proliferation of myofibroblasts can also contribute to disrupted cardiac conduction through their paracrine activity, and through physical conduction blockage by myofibroblasts [78]. With the aim of providing a model to follow changes in heart conductivity during fibrosis, Spencer et al. have proposed an in vitro fibrosis model with mathematical computing. This allowed them to demonstrate that the variation of cardiomyocyte and myofibroblast volume fractions in EHT could lead to a slower conduction velocity [79].

The heart is an organ with anisotropic qualities, which means that it shows different properties when measured in different directions. Anisotropy in a healthy heart is mediated by cardiomyocytes alignment and the structuration of the ECM. Both aspects are involved in contractile functions of the heart, and generate stronger contractile forces compared to a non-organized tissue. However, in cardiac diseases the fibrotic process disrupts this organization, leading to alteration of the cardiac structure and subsequent weaker contraction of the organ [80]. To investigate this aspect, Van Spreeuwel et al. have proposed an in vitro model exploiting neonatal mouse cardiomyocytes and CFs, seeded on collagen/Matrigel matrix, stretched with the help of a microfabricated tissue gauge. This allows the comparison of isotropic structures with anisotropic ones, making it possible to define mechanical and electrical properties of fibrotic de-structured cardiac patches versus healthy organized ones. This model allowed the researchers to demonstrate that ECM de-structuration has a negative effect on the direction and homogeneity of contractile forces caused by differences in cell shape and sarcomere organization [81].

Albeit still very restricted in their applications, future developments in these integrated designs, where biological cues meet and affect biomechanical features, will need to be strongly implemented. In fact, it has become increasingly clear that these features are tightly interconnected in fibrosis development and progression.

## 9. Conclusions

Multicellular 3D models for the study of cardiac fibrosis encompass a wide variety of options. The possibility of using human iPSC-derived differentiated cells in these complex setups brings ex vivo research to a new and exciting level of biomedical significance. The integration of multiple cell types is of paramount importance for physiologically and translationally relevant modelling of tissues. Indeed, the reciprocal intercellular conditioning of stromal, parenchymal, and (possibly) immune cells can be studied through many different setups. Some valorize mainly biological cues and spontaneous 3D architecture (e.g., spheroids/organoids); others highlight the role of the ECM (e.g., engineered tissues), or of biophysical and mechanical cues (e.g., Biowires, organs-on-chip). Nonetheless, all of them have enabled the elucidation of highly specific molecular mechanisms that would have been impossible, or at least very difficult, to identify, modulate, or analyse directly in situ. Future developments will certainly reduce the “reality gap” of these models even further.

## Data Availability

Not applicable.
